# Co-expression of tissue factor, TROP2, and NECTIN4 in primary and matched metastatic cervical cancer lesions

**DOI:** 10.1016/j.tranon.2025.102453

**Published:** 2025-06-26

**Authors:** Marit L. Ulvang, Oda Fløtre Kvile, Hege F. Berg, Kathrine Woie, Ingfrid S. Haldorsen, Alessandro D. Santin, Bjørn I. Bertelsen, Camilla Krakstad, Mari Kyllesø Halle

**Affiliations:** a Centre for Cancer Biomarkers, Department of Clinical Science, University of Bergen, Bergen, Norway; b Department of Obstetrics and Gynecology, Haukeland University Hospital, Bergen, Norway; c Mohn Medical Imaging and Visualization Centre, Department of Radiology, Haukeland University Hospital, Bergen, Norway; d Section of Radiology, Department of Clinical Medicine, University of Bergen, Bergen, Norway; e Department of Obstetrics, Gynecology and Reproductive Sciences, Yale University School of Medicine, New Haven, CT 06510, USA; f Department of Pathology, Haukeland University Hospital, Bergen, Norway

**Keywords:** Cervical cancer, ADC target, Tissue factor, NECTIN4, TROP2, Metastatic lesions, Immunohistochemistry, Recurrent disease

## Abstract

**Background:**

Early detection of cervical cancer (CC) generally leads to favorable prognosis; however, survival rates drop significantly for late-stage or recurrent disease with limited treatment options. Antibody-drug conjugates (ADCs) show promise across multiple cancers but remain sparsely explored in CC.

**Objective:**

This study evaluates ADC target expression in primary and metastatic CC to identify potential ADC responders.

**Study Design:**

Membrane expression of tissue factor, TROP2 and NECTIN4 was investigated by immunohistochemistry in 522 primary and 194 metastatic CC lesions. Expression levels were scored according to the ASCO/CAP criteria and correlated with clinicopathological features and follow-up data.

**Results:**

Tissue factor, TROP2, and NECTIN4 membranous expression were cancer-cell specific and strong (3+) in 29 %, 37 %, and 4 %, and high (≥2+) in 48 %, 68 %, and 12 % of tumors, respectively. High TF and TROP2 expression were predominantly observed in squamous cell and adenosquamous histologies (p<0.001). Overall, 80 % of primary tumors exhibited high expression of at least one of the ADC targets. High levels of tissue factor and TROP2 were detected in 45 % and 70 % of the metastatic lesions, respectively. The proportion of lesions with high NECTIN4 expression was 12 % in primary tumors and 38 % in recurrent lesions.

**Conclusions:**

The ADC targets tissue factor and TROP2 are highly expressed in primary and metastatic CC. The prevalence of high NECTIN4 expression is modest in primary tumors but increases significantly in recurrent disease. These findings support the initiation of clinical trials to evaluate safety and efficacy profiles of ADCs targeting tissue factor, TROP2 and NECTIN4 in CC.

## Highlights

• Tissue factor and TROP2 are highly expressed in both primary and metastatic cervical cancer (n = 522).
• ADC target expression varies by histology, with the highest levels in squamous cell and adenosquamous carcinomas.
• NECTIN4 expression is low in primary tumors but significantly higher in recurrent lesions.
• Overall, 80 % of primary cervical cancer express high levels of at least one of these ADC targets.

## Introduction

Despite global efforts aiming for disease elimination, the incidence and mortality rates of cervical cancer (CC) are rising. In 2022, 661,021 women were diagnosed, and 348,189 died from the disease [1]. CC is the fourth most common cancer globally in women and is classified as a public health problem by the World Health Organization [1]. Early-stage CC has a good prognosis, but survival rates drop significantly for late-stage or recurrent disease [2], with limited treatment options. Chemoradiotherapy is the predominant treatment for advanced CC but has severe short- and long-term side effects [3]. Antibody-drug conjugates (ADCs) offer promising advancement, targeting cancer cell membrane proteins to deliver cytotoxic payloads directly to tumor cells while minimizing toxicity to normal cells. Despite a revolution in newly developed ADCs [4], only tisotumab vedotin targeting tissue factor (also known as TF) and trastuzumab deruxtecan targeting HER2 [5], are approved for late-stage CC treatment.

The ADC sacituzumab govitecan (Trodelvy®) targets trophoblast cell-surface antigen-2 (TROP2) [6] and was FDA approved for patients with metastatic or locally advanced triple-negative breast cancer (TNBC) in 2021 [7]. In cancer cells, TROP2 is involved in the PI3K/AKT and MAPK signaling pathways causing cell proliferation and growth, angiogenesis, invasion and metastasis [8]. Overexpression of TROP2 has been reported in numerous solid tumors and correlates with poor survival in breast, gastric, colorectal, oral squamous, and ovarian cancer(reviewed in [9]). The prognostic role and the tumor characteristics associated with TROP2 expression in CC are unclear, however, recent immunohistochemistry (IHC) studies have indicated high TROP2 levels [[10], [11], [12], [13]]. CC cell lines have shown sensitivity to sacituzumab govitecan *in vitro* and *in vivo* in xenografts [10]. Recently, a phase 2 study (NCT05838521) testing sacituzumab govitecan in patients with recurrent or persistent CC was launched, and study results are expected in 2028 [14].

Enfortumab vedotin (Padcev®), an ADC targeting nectin cell adhesion molecule 4 (NECTIN4), gained FDA approval for metastatic or advanced urothelial carcinoma in 2021 [15]. The clinical trial EV-301 (NCT03474107) leading to approval demonstrated prolonged survival with enfortumab vedotin (ORR of 40.6 %; median OS of 12.9 months) compared to a single-agent chemotherapy (ORR 17.9 %; median OS 9.0) [16]. NECTIN4 is a transmembrane protein mainly involved in cell-cell adhesion and mainly found in the placenta [17]. High expression of NECTIN4 has been linked to poor survival in TNBC, esophageal cancer, hepatocellular and pancreatic cancer (reviewed in [18]), yet with better survival in head and neck squamous cell carcinomas [19]. In CC, NECTIN4 has been identified as a possible target in chemotherapy-resistant metastatic cells [20,21] and recently, encouraging results from a phase I/II study investigating a novel NECTIN4 targeting ADC (9MW2821; NCT05216965) was launched [22]. Yet, a comprehensive characterization of NECTIN4 expression in CC cohorts has so far not been performed.

Tissue factor, TROP2, and NECTIN4 are emerging ADC targets in various cancers but are underexplored in CC. This study aimed to characterize their expression patterns in relation to patient outcomes and tumor characteristics in a large CC cohort (n = 522). By analyzing tumor membrane expression levels alongside clinicopathological features and disease-specific survival, we aimed to identify a patient population relevant for future ADC treatment studies. Given the unknown expression levels of ADC targets in metastatic CC, we mapped their distribution across metastatic and recurrent lesions. Understanding the concordance rate between matched primary and metastatic lesions is crucial, as it provides insights into the consistency of target expression over time and under different conditions.

## Materials and methods

### Ethical approvals

All recruited patients signed written, informed consent. Tissue samples were retrieved from Bergen Biobank for Gynecological Cancer (approved by Regional komité for forskningsetikk, Vest Norge (REK-Vest) 2014/1907 ID #6647). Clinicopathological data and follow-up information were collected from Bergen Gynecological Cancer Health Registry (approved by the Norwegian Data Inspectorate 2016/7421 and REK vest #7226). This study was approved by the Regional Ethical Committee (REK-Vest 2018/591).

### Patient population

Tissue and clinical data from consenting patients treated for CC at Haukeland University Hospital were prospectively collected in the Bergen Biobank for Gynecological Cancer and Bergen Gynecological Cancer Health Registry, respectively. Haukeland University Hospital is a referral hospital for patients in Western Norway, representing 11 % of the Norwegian population with equivalent incident rates for CC as the total Norwegian population [23]. All patients were treated according to national guidelines. Clinical data, including age at primary diagnosis, FIGO-2018 stage, recurrence and treatment, were retrieved from patient records. Vascular space invasion, depth of invasion, histological type and grade were assessed by an expert pathologist, as previously described [24]. Patients included in this study were treated for CC between May 2001 and June 2020 and had similar clinical features as the full cohort except for FIGO stage due to lack of sufficient tumor tissue for low FIGO stages (Supplementary Table 1).

### Immunohistochemistry

Tissue microarrays (TMAs) were generated from a total of 522 patients, as previously described [24]. Additionally, 55 metastatic lesions from the time of primary diagnosis and 85 lesions from recurrent disease were collected from 72 individual patients. Four of the patients had metastatic lesions available from both primary diagnosis and recurrence. For the metastatic lesions, one cylinder (1 mm) of metastatic tissue was mounted onto a recipient block. For both primary and metastatic lesions, immunohistochemistry (IHC) was performed on 4 µm TMA sections with optimized protocols for the three selected antibodies (Supplementary Table 2) and visualized and evaluated as previously described [24].

Membrane staining intensities for tissue factor, TROP2, and NECTIN4 were assessed according to the American Society of Clinical Oncology/College of American Pathologists (ACSO/CAP) criteria for breast cancer [25], which is equivalent to the HercepTest® criteria [26]. Cytoplasmic and nuclear staining were considered non-specific and excluded from the evaluation. A score of 0 was given when less than 10 % of tumor cells showed membrane staining. Scores of 1+, 2+, and 3+ were assigned when more than 10 % of cells had weak, moderate, or strong membrane staining, respectively. In the analysis, scores of 0 were classified as negative, while scores of 1+ or higher were positive. Scores of 0 and 1+ were grouped as low, and scores of 2+ and 3+ as high. M.L.U. scored all lesions. In addition, two project participants (M.L.U., O.F.K. and M.K.H.) evaluated 100 overlapping tumor lesions with tissue factor, TROP2 and NECTIN4 expression. The interobserver agreement for tissue factor, TROP2 and NECTIN4 was 0.74, 0.84 and 0.75 in four scoring groups (0, 1+, 2+ and 3+; Weighted Kappa) and 0.82, 0.82 and 0.76 in two scoring groups (low and high; Kappa). Borderline cases were jointly reviewed by the three readers (M.L.U., O.F.K. and M.K.H.).

### Statistical analyses

Statistical analyses were performed using IBM SPSS Statistics and RStudio (Version 4.3.2). Probability values (p-values) were two-sided, and statistical significance was set at p<0.05. Statistical correlations between groups with categorical values were determined using Pearson’s Chi-Square test when cell frequencies were five or more, and Fisher’s exact test for cell frequencies less than five. For non-parametric independent samples, the Mann-Whitney U test was used to compare two groups, while comparisons among three or more groups were conducted using the Kruskal-Wallis test. Patient survival curves were plotted using the Kaplan-Meier estimator, and differences in survival between groups were calculated using the Mantel-Cox log-rank test. Interobserver agreement of expression scores was assessed using Kappa analysis conducted in GraphPad (www.graphpad.com).

In accordance with the journal’s guidelines and Norwegian legislation, we will provide our data for independent analysis by a selected team by the Editorial Team for the purposes of additional data analysis or for the reproducibility of this study in other centers if such is requested.

## Results

### Tissue factor and TROP2 are highly expressed in cervical cancer

We identified membrane protein levels of tissue factor, TROP2 and NECTIN4 by IHC and evaluated expression according to the ASCO/CAP guidelines (detailed in Methods). Representative TMA cores of strong (3+), moderate (2+), weak (1+) and negative (0) staining for the three investigated proteins are presented in Supplementary Fig. 1.

**Fig. 1:**
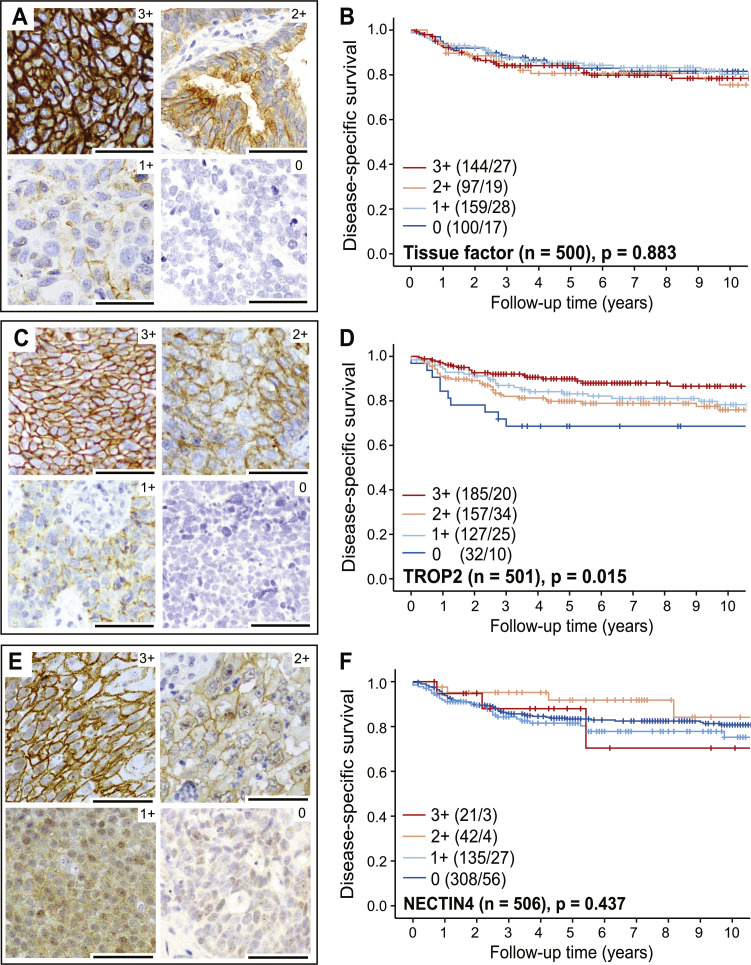
**Tissue factor, TROP2 and NECTIN4 are expressed in cervical cancer cell membranes.** Representative images of immunohistochemistry staining of tissue factor (**A**), TROP2 (**C**) and NECTIN4 (**E**) with strong (3+), moderate (2+), weak (1+) and negative (0) tumor membrane expression. Tissue factor and TROP2 expression were membrane specific, whilst for NECTIN4, also cytoplasmic staining was observed. Stroma cells were negative. All scale bars measure 100 µm. The Kaplan-Meier curves visually display probability values from comparison of tissue factor (**B**), TROP2 (**D**) and NECTIN4 (**F**) staining in four groups (0, 1+, 2+ and 3+) using the Mantel-Cox log-rank test. Parentheses contain patients/events for each category.

Tissue factor expression was strong in 29 % (n = 144), moderate in 19 % (n = 97) and weak in 32 % (n = 159) of the tumors (Fig. 1A). A total of 20 % of the tumors (n = 100) were negative for tissue factor. Tissue factor expression was not prognostic (p = 0.88) (Fig. 1B). TROP2 expression was strong in 37 % (n = 185), moderate in 31 % (n = 157), and weak in 25 % (n = 127) of the tumors (Fig. 1C). The remaining 7 % (n = 32) of the tumors were negative for TROP2. TROP2-negative tumors associated with poor survival in the full cohort (0 versus 3+, 2+, 1+; p = 0.02) (Fig. 1D) and in SCCs alone (p = 0.005) (Supplementary Fig. 2). For NECTIN4, only 4 % of the tumors (n = 21) had strong staining, 8 % (n = 42) had moderate and 27 % (n = 135) had weak staining. The remaining 61 % (n = 308) of the tumors had no membranous NECTIN4 expression (Fig. 1E). NECTIN4 expression was not prognostic (p = 0.42) (Fig. 1F).

In recent clinical trials, including those for gynecological cancer, ADCs demonstrated high response rates when the protein target expression in tumors was high (i.e., ≥2+) [27]. To identify patient subgroups eligible for ADC treatment, we compared clinicopathological features for patients with high (≥2+) versus low (≤1+) tumor ADC target expression. We found that high tissue factor expression (≥2+) associated with SCC and ASC histology (p<0.001) and low histologic grade (p = 0.04) and tended to associate with deep stromal invasion (p = 0.06) (Table 1). Similarly, high TROP2 (≥2+) expression associated with SCC, ASC and undifferentiated histology (p<0.001) and vascular space invasion (p = 0.009) and tended to associate with low histologic grade (p = 0.07) (Table 1). High NECTIN4 expression associated with SCC histology (p<0.001) and low histological grade (p = 0.03) (Table 1).

**Table 1:** Tissue factor, TROP2 and NECTIN4 membrane expression levels related to clinicopathological characteristics in cervical carcinomas.

	Tissue factor			TROP2			NECTIN4		
	Low ≤1+	High ≥2+		Low ≤1+	High ≥2+		Low ≤1+	High ≥2+	
	n ( %)		p	n ( %)		p	n ( %)		p
n total	259 (52)	241 (48)		159 (32)	342 (68)		443 (88)	63 (12)	
**Median age**	44	45	0.32	46	44	0.82	44	47	0.13
**FIGO-18**			0.27			0.19			0.72
FIGO IA	45 (62)	27 (38)		20 (27)	54 (73)		66 (86)	11 (14)	
FIGO IB	113 (52)	105 (48)		75 (34)	144 (66)		196 (89)	24 (11)	
FIGO II	29 (45)	35 (55)		24 (37)	40 (63)		53 (83)	11 (17)	
FIGO III	53 (48)	58 (52)		26 (24)	82 (76)		97 (88)	13 (12)	
FIGO IV	19 (54)	16 (46)		14 (39)	22 (61)		31 (89)	4 (11)	
**Type**			**<0.001**			**<0.001**			**<0.001**
SCC	172 (47)	191 (53)		79 (22)	287 (78)		307 (83)	62 (17)	
AC	68 (67)	34 (33)		65 (65)	35 (35)		101 (99)	1 (1)	
ASC	4 (27)	11 (73)		5 (33)	10 (67)		15 (100)	0 (0)	
NEC	10 (100)	0 (0)		8 (80)	2 (20)		10 (100)	0 (0)	
UDC	5 (62)	3 (38)		2 (25)	6 (75)		10 (100)	0 (0)	
**Grade**			**0.04**			0.07			**0.03**
G 1/2	208 (50)	212 (50)		128 (30)	297 (70)		369 (87)	57 (13)	
G 3	45 (62)	27 (38)		28 (41)	40 (59)		68 (96)	3 (4)	
**VSI**			0.16			**0.009**			0.58
No	141 (56)	112 (44)		87 (34)	170 (66)		227 (87)	35 (13)	
Yes	52 (48)	57 (52)		22 (20)	87 (80)		95 (89)	12 (11)	
**DOI**			0.06			0.85			0.06
< 7 mm	107 (61)	67 (39)		58 (33)	119 (67)		156 (86)	26 (14)	
≥ 7 mm	80 (51)	76 (49)		49 (32)	105 (68)		142 (92)	12 (8)	

All p-values are calculated by the chi-square test except for median age (Mann Whitney U test), histological type (Fisher’s Exact test) and grade for NECTIN4 (Fisher’s Exact test). Statistically significant p-values in bold (p<0.05).

Abbreviations: AC: Adenocarcinoma; ASC: Adenosquamous carcinoma; DOI: Depth of invasion; FIGO: International Federation of Gynecology and Obstetrics; G: Grade; NEC: Neuroendocrine carcinoma; SCC: Squamous cell carcinoma; UDC: Undifferentiated carcinoma; VSI: vascular space invasion.

### Most cervical carcinomas express high levels of either tissue factor, TROP2 or NECTIN4

**Fig. 2:**
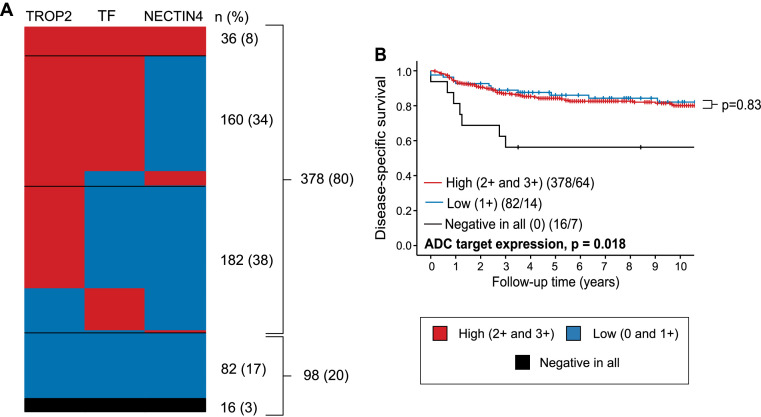
**80 % of the tumors had high membrane expression (≥2+) of at least one ADC target (i.e., TROP2, tissue factor or NECTIN4. A)** Heatmap illustrating intra-patient membrane expression levels of TROP2, tissue factor and NECTIN4 with high membrane expression (2+ and 3+) in red, low membrane expression (0 and 1+) in blue, and negative expression in all ADC targets (score 0) in black. The number and percentage of patients in each group is given on the right (n total = 476). **B**) Kaplan-Meier survival curve comparing patients with high expression of at least one of the target proteins, low expression of all proteins (≤1+) and no expression (0) of any ADC target. The Kaplan-Meier curve visually displays probability values from comparison of categories using the Mantel-Cox log-rank test. Patients/events are listed in parentheses. Abbreviation: TF: Tissue factor.

Next, we compared expression patterns across all three ADC targets in each tumor to further elucidate their clinical applicability. In total, 479 tumors were successfully stained and scored for all three target proteins. Only 8 % (n = 36) of the tumors had high expression (≥2+) of all, 34 % (n = 161) had high expression of two, and 38 % (n = 184) had high expression of one of the markers (Fig. 2A). Altogether, 80 % (n = 381) of the tumors had high expression of at least one of the target proteins, 17 % (n = 82) had low expression (1+) of all target proteins and only 3 % (n = 16) were negative for all three. While most SCCs (87 %; n = 305) and ASCs (93 %; n = 14) had a high expression of at least one target protein, most NECs (60 %; n = 6) had no expression of any target protein (Supplementary Table 3). No difference in disease-specific survival was observed between tumors with high expression in at least one target protein versus tumors with low expression of all (p = 0.83) (Fig. 2B). However, tumors negative for all target proteins were significantly associated with poor disease-specific survival (DSS) (n = 16; p = 0.02), likely driven by the large proportion of NECs in this small subgroup (Fig. 2B).

### TROP2 and tissue factor are highly expressed in metastatic lesions

ADC treatment is particularly relevant for high-risk or recurrent cervical cancer. Therefore, characterizing the prognostic value of ADC target expression in these patients is highly important. Disease-specific survival based on ADC target expression in patients with FIGO stage III, IV, and/or later recurrence is shown in Supplementary Fig. 3, with protein levels categorized into four and two groups, respectively. In this high-risk cohort, similar to the primary tumors, we find that negative or low TROP2 expression is associated with significantly poorer disease-specific survival (Supplementary Fig. 3C and 3D).

**Fig. 3:**
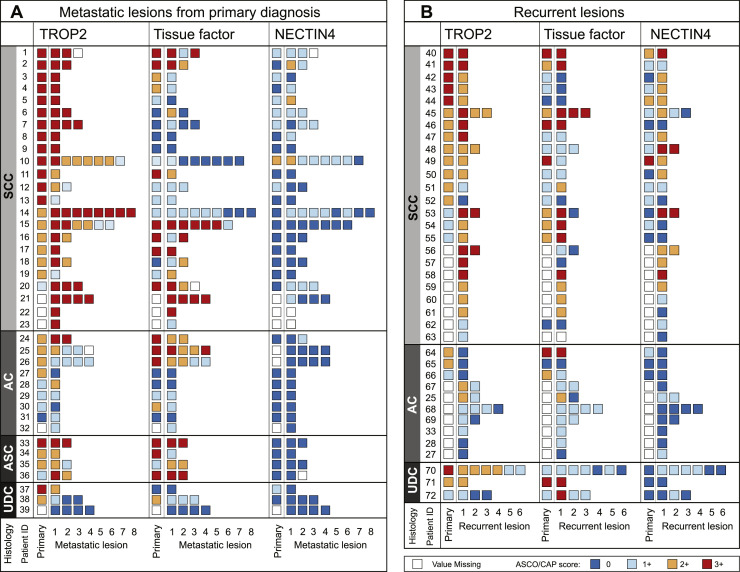
**Expression patterns of TROP2, tissue factor and NECTIN4 in primary and matched metastatic lesions from primary diagnosis (A) and recurrence (B).** Abbreviations: AC: Adenocarcinoma; ASC: Adenosquamous carcinoma; SCC: Squamous cell carcinoma; UDC: Undifferentiated carcinoma.

Given that ADCs are mostly targeting metastatic and recurrent tumors, it is crucial to know the expression levels within these metastatic lesions. To investigate the expression levels of ADC targets in the metastatic setting, we retrieved 83 metastatic lesions collected at primary diagnosis and 55 collected from later recurrence from a total of 72 individual patients. TROP2, tissue factor and NECTIN4 staining patterns in the metastatic lesions matched staining patterns observed in the primary tumors (Supplementary Fig. 4). When comparing intra-patient ADC target expression in primary versus matched metastatic lesions, high concordance in expression levels were found for both the primary metastatic (Fig. 3A) and recurrent (Fig. 3B) lesions. The level of concordance was highest for NECTIN4 (81 %; n = 47), followed by tissue factor (77 %; n = 44) and TROP2 (73 %; n = 41) when analyzed in two scoring groups (0, 1+, 2+, 3+) as well as in four scoring groups (56 % (n = 30); 54 % (n = 31); and 41 % (n = 23), respectively) (Supplementary Table 4).

Similar levels of high TROP2 expression (≥2+) were found in the metastases from primary diagnosis (76 %; n = 29), recurrent lesions (65 %; n = 24) and primary tumors (68 %; n = 342) (p = 0.25) (Supplementary Table 5). However, for tissue factor, the prevalence of high expression decreased significantly from 61 % in the primary tumors to 46 % in the primary metastases and 44 % in the recurrent lesions (p = 0.01) (Supplementary Table 5). Lesions from primary metastases had comparable levels of high NECTIN4 expression as the primary tumors (8 % versus 12 %), yet the recurrent lesions had significantly higher expression (38 %; n = 14) (p<0.001) (Supplementary Table 5).

**Fig. 4:**
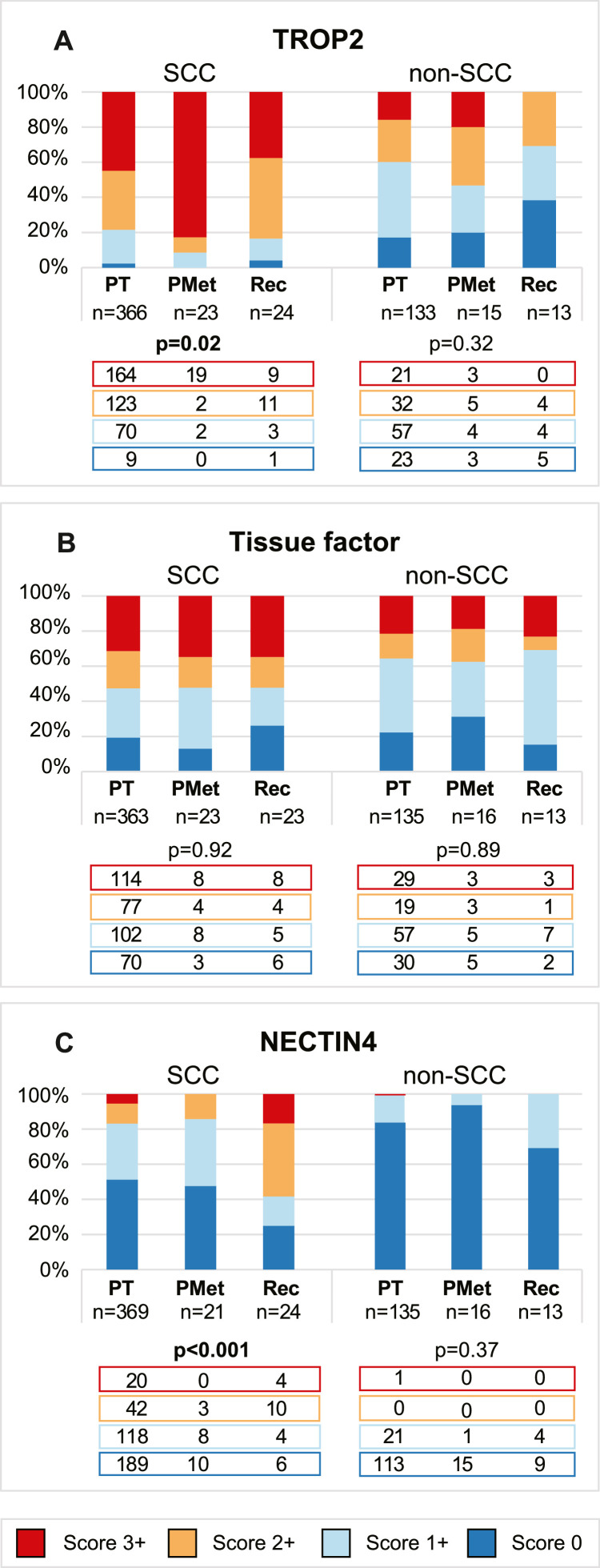
**High expression (≥2+) of TROP2 and tissue factor is maintained in the metastatic setting**. Expression levels of TROP2 (**A**), tissue factor (**B**) and NECTIN4 (**C**) in the full cohort of primary tumors (PT), primary metastases (PMet) and recurrent lesions (Rec) in squamous cell carcinoma (SCC*, left)* and non-SCC *(right).* Numbers of patients with tumor expression scores of 3+ (*red*), 2+ (*yellow*), 1+ (*light blue*) and 0 (*blue*) are displayed beneath each color bar. All p-values are calculated by the Fisher’s exact if less than 5 and indicated by bold if significant. Abbreviations: PMet: Primary metastasis; PT: Primary tumor; Rec: Recurrent lesion; SCC: Squamous cell carcinoma.

Considering the histology-dependent ADC target expression in primary tumors, we compared expression levels between primary and metastatic lesions in SCC and non-SCC subgroups, respectively (Fig. 4). Among the SCCs, the prevalence of high TROP2 expression increased significantly from 78 % (n = 287) in the primary tumors to 91 % (n = 21) and 83 % (n = 20) in the primary metastases and recurrent lesions, respectively (p<0.001) (Fig. 4A). In the non-SCC tumors, the prevalence of high TROP2 expression was 40 % (n = 53) in the primary tumors, 53 % (n = 8) in the metastatic lesions from primary diagnosis and 31 % (n = 4) in recurrent lesions (p = 0.01) (Fig. 4A). The prevalence of high tissue factor expression was similar across primary, primary metastatic, and recurrent lesions in both SCCs (p = 0.14) (Fig. 4B) and non-SCCs (p = 0.12) (Fig. 4B). For NECTIN4, high expression in the SCCs was observed in 17 % (n = 62) of the primary tumors and 14 % (n = 3) of the primary metastases. In contrast, 67 % (n = 14) of recurrent SCC lesions showed high NECTIN4 expression (p<0.001) (Fig. 4C). Among non-SCCs, only one primary tumor (<1 %) and none of the primary metastases or recurrent lesions had high NECTIN4 expression, yet the prevalence of tumors scoring 1+ was significantly highest in the recurrent lesions (p = 0.01) (Fig. 4C).

## Comment

### Summary of main results

In this study, we investigated expression levels of three promising ADC targets in a large population-based cervical cancer (CC) cohort, aiming to reveal new treatment opportunities. We find that 80 % of CC patients have high levels of one or more of these ADC targets (≥2+) and are thus potential responders to ADC treatment targeting tissue factor, TROP2 or NECTIN4. By lowering the inclusion criteria to 1+ (i.e., low expression, as recently proven successful in a clinical trial targeting HER2 including cervical carcinomas among others [28]), 97 % of the patients are eligible for these ADC treatments. Notably, by only considering ADCs targeting TROP2, 93 % or 68 % of the patients could be candidates when using inclusion criteria of ≥1+ or ≥2+, respectively. Interestingly, we find particularly high ADC target expression within both squamous cell and adenosquamous tumors. Currently, no curative treatment exists for metastatic and recurrent CC [29]. This study reveals high levels of TROP2 and tissue factor also in the metastatic and recurrent lesions, indicating that the presented ADC targeting strategies are promising for CC patients with metastatic and recurrent disease.

### Results in the context of published literature

We observed positive tissue factor expression (≥1+) in 80 % and high expression (≥2+) in 48 % of the primary tumors. Tissue factor is a membrane bound surface protein involved in blood coagulation, angiogenesis, tumor growth and metastasis [30] and has been linked to poor prognosis in several solid tumors, including colorectal [31], gastric [32], bladder [33], breast [34], prostate [35], hepatocellular [36] and pancreatic ductal cancer [36]. Yet, we find no link to poor survival for CC patients. In two previous studies, high tissue factor expression was reported in 34 % and 77 % of the cervical carcinomas [37,38]. According to our data, TROP2 is even more frequently expressed in CC than tissue factor – with positive (≥1+) expression in 93 % and high expression (≥2+) in 68 % of the tumors, similar to previous reports [[10], [11], [12]]. The prognostic role of TROP2 in CC has been inconclusive [12,13]. In our cohort, comprising the largest CC cohort investigating TROP2 to date (n = 501), negative TROP2 expression associated with poor survival. In line with a previous study on TROP2 [12], we found the highest TROP expression levels within the SCCs and ASCs. Together, our and previous studies indicate that TROP2 may be highly relevant ADCs target in CCs – particularly in SCCs and ASCs.

To our knowledge, this is the first study evaluating NECTIN4 expression in CC. We found positive NECTIN4 expression (≥1+) in 39 % and high expression (≥2+) in 12 % of the primary tumors. Except for one AC case, high NECTIN4 expression was detected exclusively in SCCs (17 %, n = 62). NECTIN4 expression is observed in several other cancer types predominated with squamous histology with prevalence of high expression (moderate/strong) varying from 60 % in bladder cancer [39], 33 % in head and neck tumors [19], to 11 % of penile cancer [40]. In line with another study investigating metastatic lung, ovarian and breast cancer [39], we find higher NECTIN4 levels in the recurrent lesions. This points to NECTIN4 targeting as a plausible strategy, particularly within recurrent SCCs.

### Clinical relevance of or findings

Identifying ADC target expression in rare disease is of high clinical relevance, as these tumors are highly aggressive and respond poorly to current treatments [41,42]. In line with a previous study in neuroendocrine CC [43], we find modest expression of ADC targets in neuroendocrine CC. However, in the ASCs and UDCs, we found high expression of TROP2 and tissue factor. Our discovery of distinct expression patterns in relation to histology may help clinicians identify targeted treatment strategies for patients with rare CC histologies. However, the small sample sizes necessitate multi-institutional studies for validation across diverse populations and histologies.

We found significant expression of tissue factor and TROP2 in both primary and metastatic lesions, which is promising for future cervical cancer patients. ADCs have so far been minimally explored in CC patients [4], thus there is a significant potential for refining treatment protocols. New clinical trials, such as phase 1/2 trials testing ADCs targeting TROP2 (LCB84 with a PD-1 inhibitor; NCT05941507) and NECTIN-4 (single-agent LY4101174; NCT06238479), are underway. Optimizing response rates depends on refining predictive biomarkers, staining protocols, and cut-offs. The emerging role of PET tracers to noninvasively detect ADC target levels in tumors (e.g., 68Ga-THP-Trop2 in NCT06465017) will likely enhance patient stratification. These aspects need further testing in clinical trials involving cervical cancer patients.

### Strengths and weaknesses

This population-based cohort study is distinguished by its extensive patient inclusion, surpassing previous studies. It also benefits from expert pathology review of all included tumor samples, ensuring high accuracy and reliability of the findings.

As for all biomarker studies, standardization of staining protocols including antigen retrieval, blocking, choice of antibody and incubation time is crucial for clinical implementation. To further evaluate the predictive power of the proposed ADC response biomarkers, their expression cut-offs and staining protocols need to be validated in cohorts with response data obtained from clinical trials.

## Conclusions

Tissue factor and TROP2 are highly expressed in primary, metastatic and recurrent CCs. SCCs express high levels of all three ADC targets while ASCs show high tissue factor and TROP2 levels. Overall, TROP2 was the most prevalent of the ADC targets investigated. This was maintained in the metastatic lesions. For NECTIN4, higher expression was detected in recurrent disease compared to primary tumors. Clinical trials evaluating the safety and efficacy of ADCs are highly relevant in CC.

## Additional information

### Funding information

This study was supported by the University of Bergen; Norwegian Cancer Society (grants 104484, 190202 & 223283); the Research Council of Norway (grants 326348 and 273280); and Western Norway Regional Health Authority (HelseVest) (grant F12542). None of the funders was involved in the study design, data collection, data analysis, interpretation or writing of the manuscript. This study was performed in accordance with the Declaration of Helsinki.

### Declaration of interest statement

The authors declare that they have no known competing financial interests or personal relationships that could have appeared to influence the work reported in this paper.*

### Declaration of generative AI and AI-assisted technologies in the writing process

During the preparation of this work the authors used Microsoft Co-pilot in order to improve language and readability. After using this tool/service, the authors reviewed and edited the content as needed and take full responsibility for the content of the publication.

## CRediT authorship contribution statement

**Marit L. Ulvang:** Formal analysis, Investigation, Methodology, Software, Validation, Visualization, Writing – original draft, Writing – review & editing, Conceptualization. **Oda Fløtre Kvile:** Formal analysis, Investigation, Methodology, Validation, Writing – original draft, Writing – review & editing. **Hege F. Berg:** Formal analysis, Methodology, Validation, Writing – review & editing. **Kathrine Woie:** Data curation, Supervision, Validation, Writing – review & editing. **Ingfrid S. Haldorsen:** Data curation, Funding acquisition, Resources, Supervision, Writing – review & editing, Methodology. **Alessandro D. Santin:** Investigation, Methodology, Supervision, Writing – review & editing. **Bjørn I. Bertelsen:** Data curation, Investigation, Methodology, Supervision, Validation, Writing – review & editing, Formal analysis. **Camilla Krakstad:** Data curation, Funding acquisition, Methodology, Project administration, Resources, Supervision, Validation, Writing – review & editing, Conceptualization, Visualization, Writing – original draft. **Mari Kyllesø Halle:** Conceptualization, Data curation, Formal analysis, Funding acquisition, Methodology, Project administration, Resources, Software, Supervision, Validation, Visualization, Writing – original draft, Writing – review & editing.

## Declaration of competing interest

The authors report no conflict of interest.

## Appendix Supplementary materials

Image, application 1
