# Supplementary material for: Co-expression of tissue factor, TROP2, and NECTIN4 in primary and matched metastatic cervical cancer lesions
Source: Transl Oncol. 2025 Jun 26;59:102453. doi: 10.1016/j.tranon.2025.102453 (PMC12302420; doi:10.1016/j.tranon.2025.102453)
Supplement: Supplementary file 1 [file mmc1.pdf]

Supplementary Material

Tissue factor, TROP2, and NECTIN4 expression in Primary and Metastatic Cervical Cancer

Marit L. Ulvang[1,2], Oda Fløtre[1,2], Hege F. Berg[1,2], Kathrine Woie[2], Ingfrid S. Haldorsen[3,4], Alessandro D. Santin[5], Bjørn I. Bertelsen[6], Camilla Krakstad[1,2,$], Mari Kyllesø Halle[1,2,$*]

[1]Centre for Cancer Biomarkers, Department of Clinical Science, University of Bergen, Bergen, Norway.
[2]Department of Obstetrics and Gynecology, Haukeland University Hospital, Bergen, Norway.
[3]Mohn Medical Imaging and Visualization Centre, Department of Radiology, Haukeland University Hospital, Bergen, Norway.
[4]Section of Radiology, Department of Clinical Medicine, University of Bergen, Bergen, Norway.
[5]Department of Obstetrics, Gynecology and Reproductive Sciences, Yale University School of Medicine, New Haven, CT 06510, USA
[6]Department of Pathology, Haukeland University Hospital, Bergen, Norway.
[$]shared last authors.

Table of content

Supplementary Table 1 2
Supplementary Table 2 2
Supplementary Table 3 3
Supplementary Figure 1.. 4
Supplementary Figure 2 5
Supplementary Figure 3 6
Supplementary Table 4 7
Supplementary Table 5 7

Supplementary Table 1: Comparison of available clinicopathological characteristics in the full cohort and the study population.

Variables	Full cohort, n (%)	Study population, n (%)	P value[a]
n, total (%)	n = 948	n = 522	
**Median Age (range)**	**n = 948**	**n = 522**	0.27[b]
	44 (22-95)	45 (22-95)	
**FIGO-18**	**n = 948**	**n = 522**	**< 0.01**
FIGO IA	260 (27)	79 (15)	
FIGO IB	329 (35)	228 (44)	
FIGO II	119 (13)	66 (12.5)	
FIGO III	175 (18)	113 (21.5)	
FIGO IV	65 (7)	36 (7)	
**Histology type**	**n = 855**	**n = 522**	0.51
Squamous cell carcinoma	613 (72)	380 (73)	
Adenocarcinoma	199 (23)	107 (20)	
Adenosquamous carcinoma	18 (2)	15 (3)	
Neuroendocrine carcinoma	15 (2)	10 (2)	
Undifferentiated carcinoma	10 (1)	10 (2)	
**Histologic grade**	**n = 703**	**n = 513**	0.45
Grade 1 and 2	589 (84)	438 (85)	
Grade 3	114 (16)	75 (15)	
**Recurrence**	**n = 946**	**n = 521**	0.80
No	799 (85)	435 (84)	
Yes	107 (11)	65 (12)	
Metastatic at primary	40 (4)	21 (4)	

[a] Chi-squared test; [b] Mann-Whitney U test
Abbreviations: FIGO: International Federation of Gynecology and Obstetrics

Supplementary Table 2: Staining protocol for immunohistochemistry.

**Protein target**	**Antigen retrieval**	**Primary antibody**	**Antibody dilution**	**Provider**	**Incubation time in RT**
Tissue factor	Tris EDTA pH9 (S2367, Dako, Denmark)	Anti-Tissue Factor [EPR22548-232]; AB228968; RRID: AB_2904202	1:200	Abcam, Cambridge, UK	1h
TROP2	Tris EDTA pH9 (S2367, Dako, Denmark)	Anti-TROP2 [SP294] (AB227690)	1:400	Abcam, Cambridge, UK	1h
NECTIN4	Tris EDTA pH6 (S1699, Dako, Denmark)	Recombinant Anti-Nectin-4 antibody [EPR15613-68] (AB192033)	1:500	Abcam, Cambridge, UK	1h

Abbreviations: EDTA: Ethylenediaminetetraacetic acid; RT: Room Temperature; UK: United Kingdom.
RRIDs Providers: DAKO: RRID: SCR_013530; Abcam: RRID: SCR_012931

Supplementary Table 3: Correlation between clinicopathological variables and combined expression pattern of TROP2, tissue factor and NECTIN4 in 479 cervical carcinomas.

**Variables (n)**	**High in min one, (≥2+) n (%)**	**Low in all, (1+) n (%)**	**Negative in all, (0) n (%)**	**P value**
n, total (%)	n = 381 (80)	n = 82 (17)	n = 16 (3)	
**Median age (n = 479)**	47	44	45	0.743[a]
**FIGO-18 (n = 479)**				0.187[b]
FIGO IA	53 (76)	17 (24)	0 (0)	
FIGO IB	166 (80)	35 (17)	7 (3)	
FIGO II	47 (76)	12 (19)	3 (5)	
FIGO III	90 (86)	11 (10)	4 (4)	
FIGO IV	25 (74)	7 (21)	2 (6)	
**Histology type (n = 477)**				**<0.001[b]**
SCC	305 (87)	39 (11)	7 (2)	
AC	52 (56)	38 (41)	3 (3)	
ASC	14 (93)	1 (7)	0 (0)	
NEC	2 (20)	2 (20)	6 (60)	
UDC	6 (75)	2 (25)	0 (0)	
**Histologic grade (n = 471)**				**<0.001[c]**
Grade 1 and 2	331 (82)	66 (16)	6 (2)	
Grade 3	45 (66)	13 (29)	10 (15)	

[a]Kruskal-Wallis Test; [b]Fisher's exact test; [c]Chi-squared test.

Abbreviations: FIGO: International Federation of Gynecology and Obstetrics. SCC: Squamous cell carcinoma, AC: Adenocarcinoma, ASC: Adenosquamous carcinoma, NEC: Neuroendocrine carcinoma, UDC: Undifferentiated carcinoma

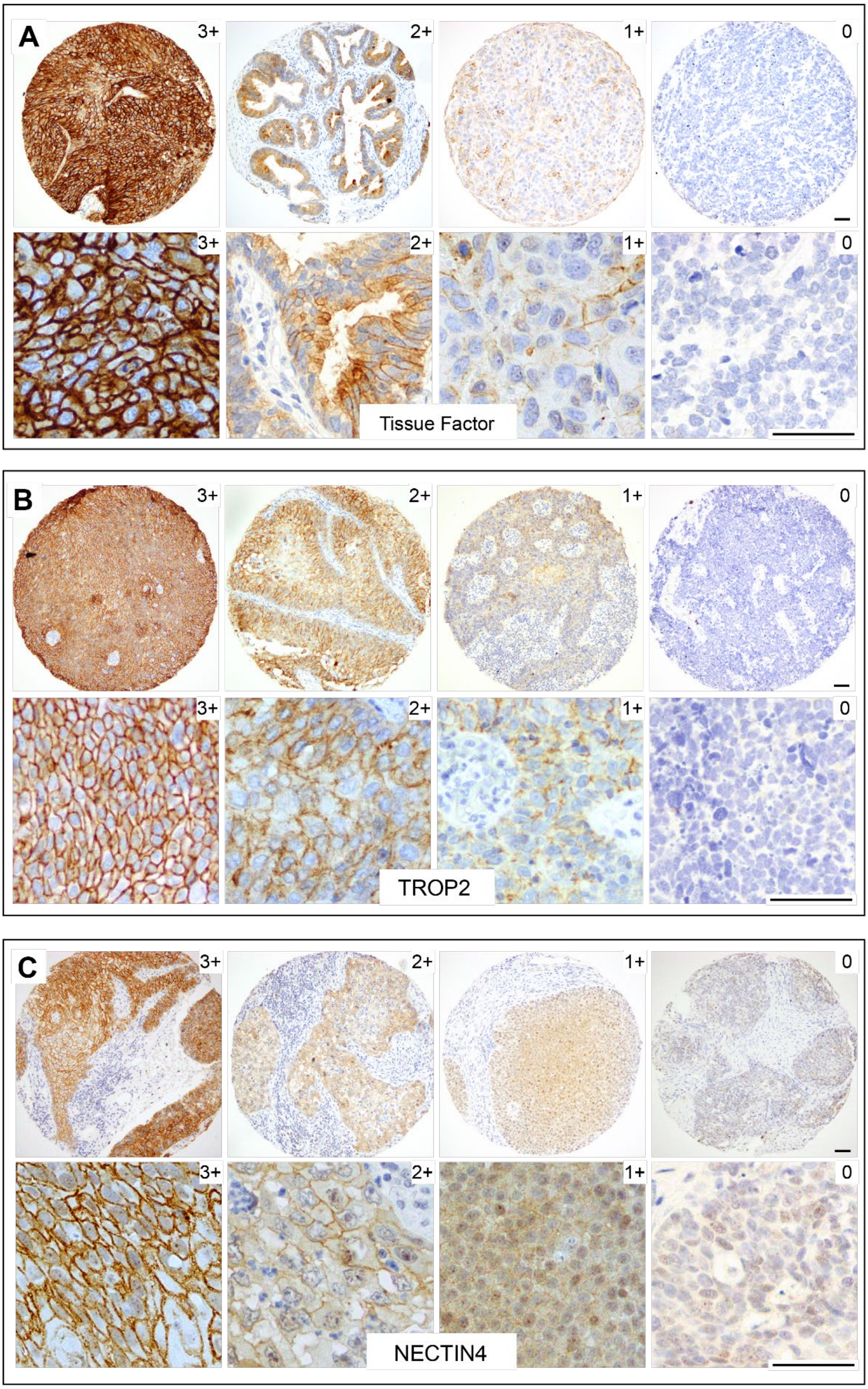

Supplementary Figure 1: ADC target proteins are clearly located in the cell membrane of cervical cancers. Representative images of immunohistochemistry staining of tissue factor **(A)** TROP2 **(B)** and NECTIN4 **(C)** with expression score from strong (3+), to moderate (2+), weak (1+) and negative (0) in tumor tissue. All scale bars measure 100 µm.

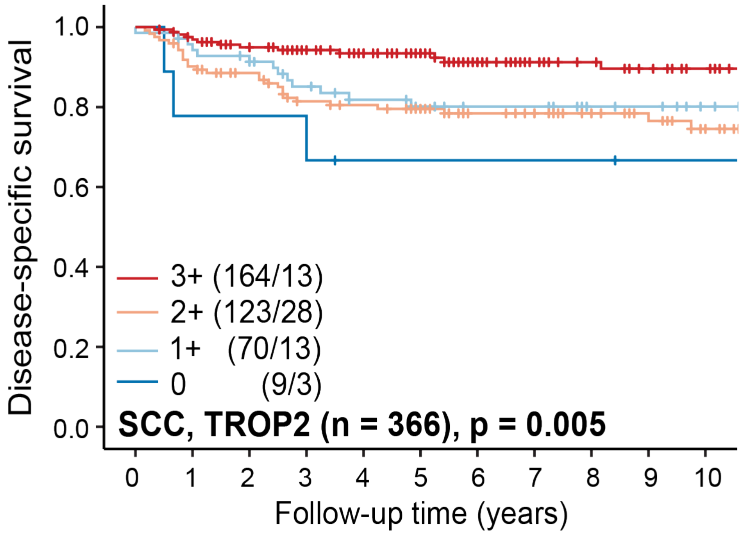

Supplementary Figure 2. TROP2 negative (0) expression associates with poor disease specific survival within patients with SCC. The Kaplan-Meier curves visually display probability values from comparison of TROP2 staining in four groups (0, 1+, 2+ and 3+) using the Mantel-Cox log-rank test. Parentheses contain patients/events for each category. Abbreviations: SCC: Squamous cell carcinoma.

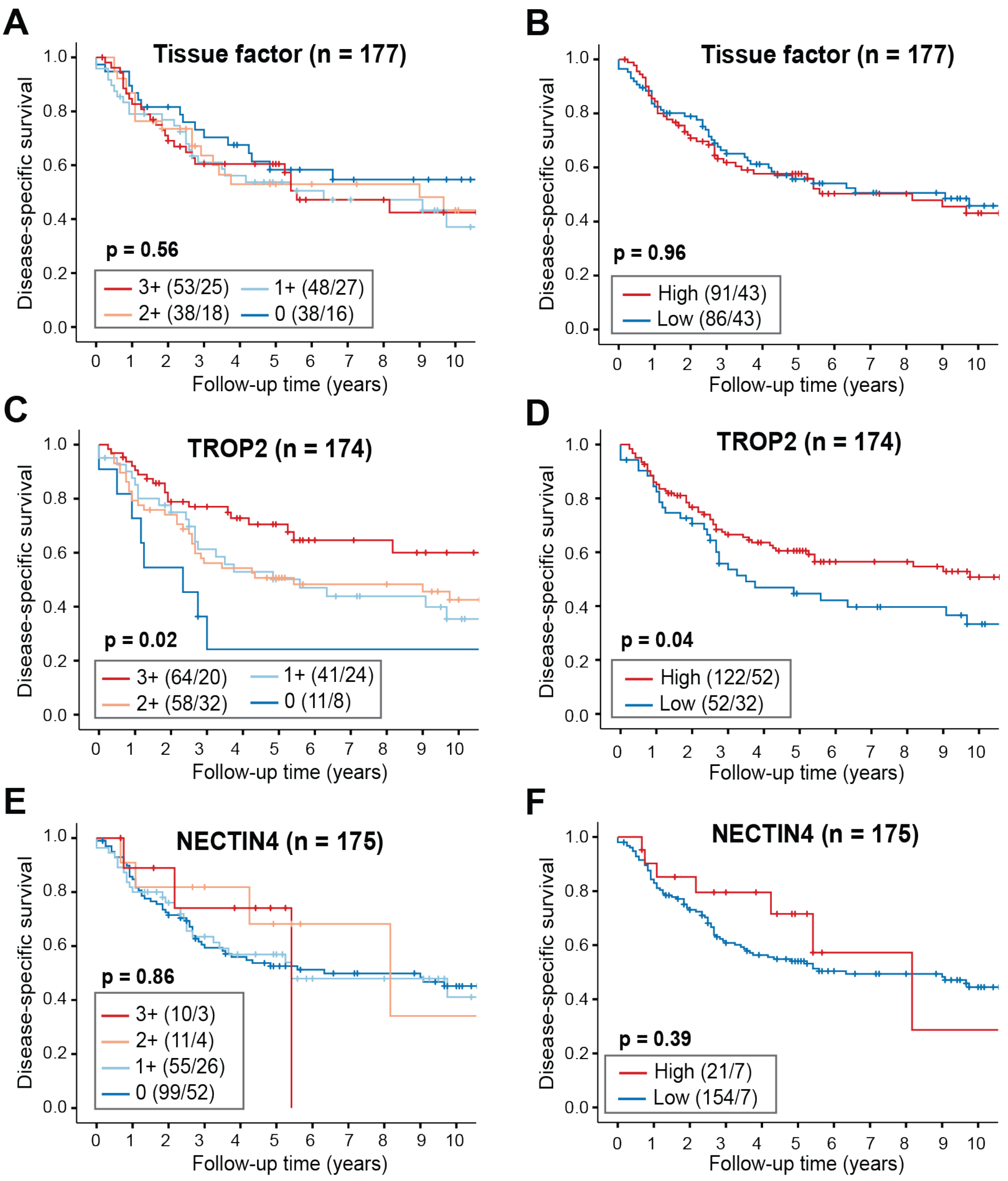

Supplementary Figure 3. Similar patterns of prognostic relevance of the three markers in the high-risk cohort as compared to the whole cohort. The Kaplan-Meier curves visually display probability values from comparison of tissue factor (**A** and **B**), TROP2 (**C** and **D**) and staining in four (0, 1+, 2+ and 3+) (**A**, **C** and **E**) and two (i.e., high: ≥2+ and low ≤1+) (**B**, **D** and **F**) groups using the Mantel-Cox log-rank test. Parentheses contain patients/events for each category.

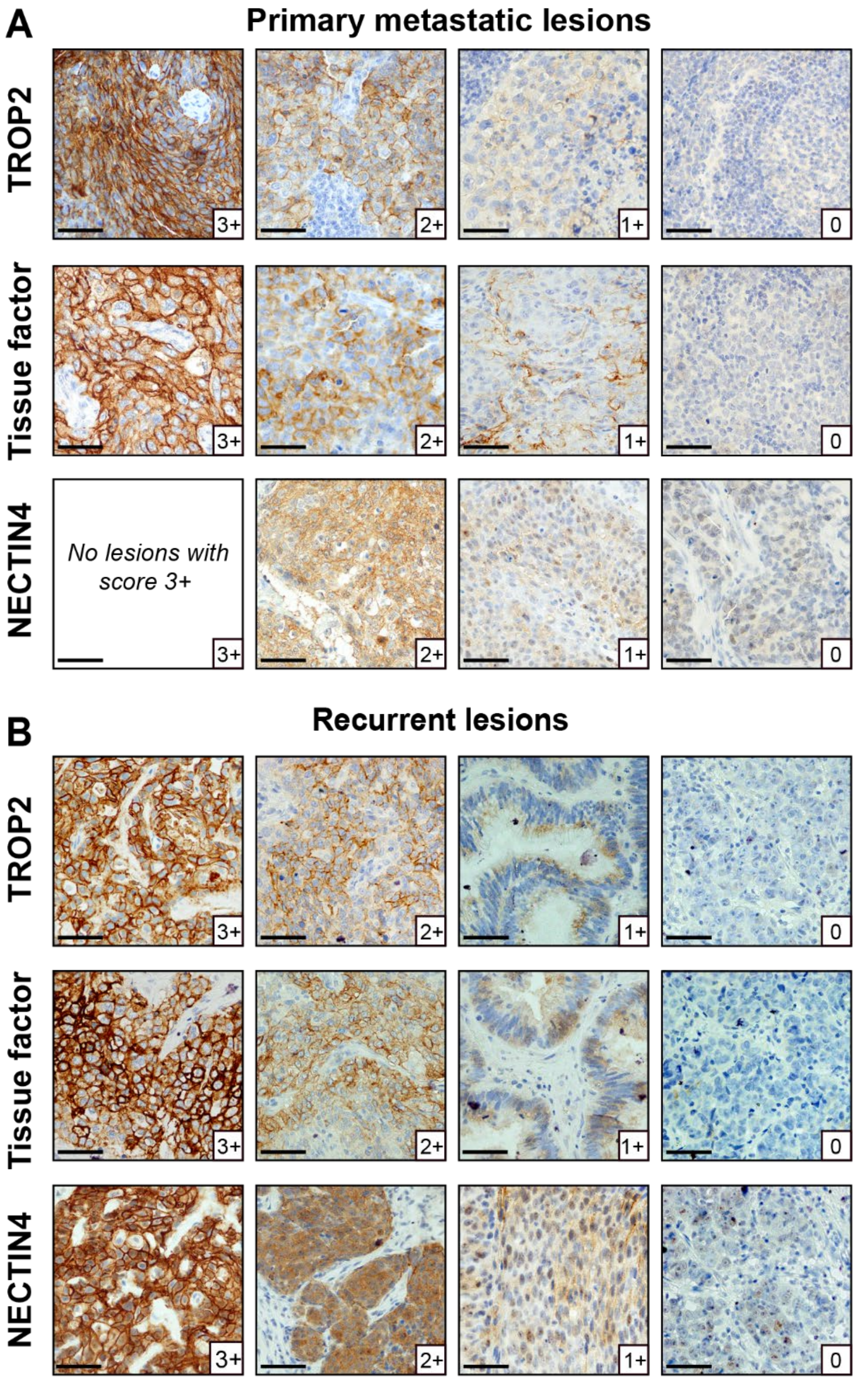

Supplementary Figure 4: Metastatic lesions display similar expression patterns of TROP2, tissue factor and NECTIN4 as lesions from primary diagnostics. Immunohistochemical staining of TROP2, tissue factor and NECTIN4 in primary metastases (**A**) and recurrent lesions (B). TROP2 and tissue factor have membrane specific protein expression. For NECTIN4, also cytoplasmic staining was observed. All scale bars measure 50 µm.

Supplementary Table 4: Number and percentage of patients with concordant TROP2, tissue factor and NECTIN4 expression in primary and matched metastatic lesions.

ADC target	Concordance 2 groups		Concordance 4 groups	
	n	*%*	*n*	*%*
TROP2	41	73	23	41
Tissue factor	44	77	31	54
NECTIN4	44	81	30	56

Abbreviations. ADC: Antibody-drug conjugate.

Supplementary Table 5. Number and percentage of TROP2, Tissue factor and NECTIN4 expression in patients with metastatic (at primary diagnosis) and recurrent lesions from cervical cancer.

	TROP2, n (%)			Tissue factor, n (%)			NECTIN4, n (%)		
**IHC score**	**PT**	**Pmet**	**Rec**	**PT**	**Pmet**	**Rec**	**PT**	**Pmet**	**Rec**
	n = 501	n = 38	n = 37	n = 500	n = 39	n = 36	n = 506	n = 38	n = 37
		**p<0.001**			**p<0.001**			**p<0.001**	
3+	185 (37)	22 (56)	9 (24)	144 (29)	11 (28)	11 (31)	21 (4)	0 (0)	4 (11)
2+	157 (31)	7 (18)	15 (41)	159 (32)	7 (18)	5 (14)	42 (8)	3 (8)	10 (27)
1+	127 (25)	6 (18)	7 (19)	97 (19)	13 (33)	12 (33)	135 (27)	9 (24)	8 (22)
0	32 (7)	3 (8)	6 (16)	100 (20)	8 (21)	8 (22)	308 (61)	25 (68)	15 (40)
		p=0.25			**p=0.01**			**p<0.001**	
High (2+ and 3+)	342 (68)	29 (76)	24 (65)	303 (61)	18 (46)	16 (44)	63 (12)	3 (8)	14 (38)
Low (0 and 1+)	159 (32)	9 (24)	13 (35)	197 (39)	21 (54)	20 (56)	443 (88)	34 (92)	23 (62)

Abbreviations: IHC: Immunohistochemistry, PT: Primary tumor, Pmet: Primary metastatic lesion, Rec: Recurrent lesion
